# Self-testing for 5 respiratory viruses in adult VACCELERATE volunteers in Germany—a pilot study on multi-pathogen rapid antigen testing to monitor community-acquired acute respiratory infections

**DOI:** 10.3389/fpubh.2025.1638280

**Published:** 2025-09-03

**Authors:** Julia A. Nacov, Jon Salmanton-García, Sarah Grimm, Fiona A. Stewart, Louise M. Cremer, Lisa Marie Rochel, Erik Müller, Carolin Joisten, Christina Többen, Ben Mechtel, Julian Fleig, Ullrich Bethe, Sibylle C. Mellinghoff, Zoi D. Pana, Heinz-Josef Schmitt, Oliver A. Cornely, Jannik Stemler

**Affiliations:** ^1^Faculty of Medicine and University Hospital Cologne, Institute of Translational Research, Cologne Excellence Cluster on Cellular Stress Responses in Aging-Associated Diseases (CECAD), University of Cologne, Cologne, Germany; ^2^Department I of Internal Medicine, Faculty of Medicine and University Hospital Cologne, Excellence Center for Medical Mycology (ECMM), University of Cologne, Cologne, Germany; ^3^German Centre for Infection Research (DZIF), Partner Site Bonn-Cologne, Cologne, Germany; ^4^Faculty of Medicine and University Hospital Cologne, Clinical Trials Centre Cologne (ZKS Köln), University of Cologne, Cologne, Germany; ^5^Medical School, University of Nicosia (UNIC), Nicosia, Cyprus

**Keywords:** incidence, point prevalence, surveillance, SARS-CoV-2, influenza, RSV, ADV

## Abstract

**Background:**

Acute respiratory infections (ARI) are the most common human infections. Diagnostic testing for respiratory pathogens is largely restricted to medical institutions. Self-testing may allow for real-time epidemiological monitoring of ARI pathogens, in particular in individuals not seeking medical attention.

**Methods:**

Adults from the VACCELERATE Volunteer Registry received a test-kit for multiple respiratory antigens, a laminar flow device (LFD) detecting severe acute respiratory syndrome coronavirus 2 (SARS-CoV-2), influenza viruses A and B, respiratory syncytial virus (RSV) and adenovirus (ADV) from a single nasal swab. During one winter season, participants self-tested upon ARI symptoms, reported LFD result as well as symptoms, vaccination history, and any underlying conditions. Participants who remained without ARI symptoms self-tested on the last day of the study period.

**Results:**

Between December 7, 2022, and June 2, 2023, 1,429 participants communicated their self-testing results. Of these 1,119 (78.3%) individuals had developed ARI symptoms and self-tested until May 31, 2023. Overall, 359 of 1,119 (32.1%) tests were positive with 17 (1.5%) co-detections, resulting in 377 detected pathogens overall. Thirteen tests (1.2%) were invalid or failed technically, and 747 (66.8%) were negative. The most frequently detected pathogen was SARS-CoV-2 (*n* = 178; 47.2%) followed by RSV (*n* = 109; 28.9%) and influenza A virus (*n* = 74; 19.6%). ADV was detected in 10 (2.7%) and influenza B virus in six (1.6%) samples only. Participants with detection of influenza A virus reported more often cough (91%), fever (59.7%), and myalgia (43.3%) than participants with detection of RSV or SARS-CoV-2. The remaining 310 participants (21.7%) who had not developed ARI symptoms self-tested on June 1, 2023, yielding an RSV point prevalence of 7.1%.

**Conclusion:**

This study allowed real-time reporting of five endemic ARI pathogens in a citizen science project. Symptom burden was highest in influenza A. Seasonal and off-seasonal RSV detection hint toward relevant RSV circulation in adults all year round.

## Introduction

Acute respiratory infections (ARI) are among the most common infections in humans (1). They are caused by a variety of airborne pathogens including severe acute respiratory syndrome coronavirus 2 (SARS-CoV-2), influenza A and B virus, respiratory syncytial virus (RSV), and many others (2). Early detection of underlying pathogens has implications for both clinical management and surveillance, permitting timely initiation of treatment, if indicated, and for infection prevention and control measures (3, 4). Ascertaining etiology mainly relies on antigen or nucleic acid amplification tests (NAAT) (5). Testing is largely restricted to hospitals or medical laboratories in light of technical requirements and cost, and therefore mostly reserved to inpatients or risk groups (6, 7).

Surveillance systems for tracking seasonal influenza and COVID-19 are implemented in many countries (8). In the United States, community surveillance for a large panel of respiratory viruses has been reported in a small sample (9). In Germany, the Robert Koch-Institute records laboratory-confirmed and otherwise reported ARI (10, 11). At a population level, online and web-based monitoring systems involving members of the general public have been broadly established for live reporting of symptoms of respiratory infections and other infectious diseases (12–14). However, actual testing for ARI in the general population is done to a very limited extent only, as laboratory capacities are limited and cost of molecular testing is considerable (6, 7, 15). The true incidence of infections and thus the natural disease course of ARI pathogens—either symptomatic or asymptomatic—remains largely unknown (11, 15, 16).

During the COVID-19 pandemic, rapid antigen testing for SARS-CoV-2 was adopted by the general public. Rapid antigen testing provides high specificity, acceptable sensitivity, and instant availability of results, all at low cost (17, 18). Recently, multiplex assays for simultaneous detection of multiple viruses from one respiratory sample became commercially available (5). The MAK5 laminar flow device (LFD; BioTeke, Wuxi, China) detects the following viruses: SARS-CoV-2, influenza A and B, RSV and adenovirus (ADV).

## Methods

### Study design and population

For this prospective nation-wide citizen science study, the VACCELERATE Volunteer Registry was used, approved by the Ethics Committee of the Medical Faculty of the University of Cologne, Germany (identifier 20–1536) (19). Electronic informed consent was obtained from all participants prior to enrolment. All research was performed in accordance with ethical guidelines and the Declaration of Helsinki.

By accessing the website https://vaccelerate.eu/volunteer-registry-2, European volunteers sign up via an electronic survey signaling interest in participating in clinical studies and scientific projects (19). By November 2022, over 30,000 adult volunteers throughout Germany had registered in the VACCELERATE Volunteer Registry.

A random sample of 10,000 adult VACCELERATE volunteers was invited via e-mail to participate in the present study. The inclusion criteria were (1) residing in Germany, (2) age ≥18 years, (3) electronic informed consent to participate in the study, and (4) availability of photo uploads. Main exclusion criteria were limited capacity to give consent, and restricted capacity to self-test.

The first 1,993 volunteers who consented to participate were sent one MAK5 test kit between December 7 and 9, 2022, and 1,952 participants actually received the test kit. The test kit consisted of an immunochromatographic LFD detecting ADV, SARS-CoV-2, RSV, and influenza A and B virus from a single respiratory sample (Supplementary Table 1: Clinical performance of MAK5), together with user instructions.

### Longitudinal ARI assessment

For assessment of a first ARI episode, participants were instructed to promptly self-apply the MAK5 test between receipt of the test kit and May 31, if one or more upper (ageusia, anosmia, pharyngitis, rhinorrhea), or lower (cough, sputum production, dyspnea) respiratory and/or general symptoms (headache, conjunctivitis, fever, myalgia) were present for ≥24 h.

Test results including a photo of the LFD strip (Supplementary Figure 1) and information on onset and quality of related symptoms were self-reported by e-mail. Incoming replies were processed daily by the study team. Only the first ARI episode and related symptoms were reported as only one test kit was provided. The longitudinal assessment period ended on May 31, 2023.

### Point prevalence in individuals without ARI symptoms

For assessment of point prevalence of the different ARI pathogens, participants who had not used the test kit until May 31, 2023, were instructed to self-test on June 1, 2023, regardless of whether symptoms were present or not. Test results including a photo of the test strip and information on any symptoms were self-reported by e-mail.

### Health and immunization status

To evaluate factors impacting the occurrence of ARI, participants were asked to fill in an electronic case report form (eCRF) for collecting information on previous laboratory-confirmed ARI, and their influenza and COVID-19 vaccination history. Demographics and data on underlying chronic conditions were already documented in the VACCELERATE Volunteer Registry and were updated by participants during this evaluation as applicable.

### Statistical analysis

The data was analyzed using SPSS v27.0 (SPSS, IBM Corp., Chicago, IL, United States). Categorical data were described using frequencies and percentages, whereas continuous data were expressed as median, interquartile range (IQR) and absolute range. The incidence for each MAK5 ARI pathogen was calculated as the number of new cases divided by the number of active participants, and were provided for each calendar week (CW), a period of seven consecutive days starting as of Sunday and ending on Saturday. The number of active participants (denominator) was constantly adjusted for the calculation of incidences. Participants who had already used their test kit were excluded from the calculation. This means that the denominator in each calendar week, synonymous with the number of active participants, decreased over time. A participant still having an unused test and still in contact with the study team was considered an active participant. To account for imperfect test sensitivity, corrected incidence estimates were calculated by dividing the number of reported pathogen detections by the reported sensitivity of the MAK5 test (Supplementary Table 1). The resulting corrected number of cases was then divided by the total number of active participants in the respective calendar week to yield the corrected incidence. A comparison of proportions was performed using the chi-square test. The significance level was set at a *p*-value of <0.05. If one variable for a specific analysis had missing data in a valid case, this case was excluded from the respective analysis. A scale projection specific for Germany, as well as assessment as to the representativity in terms of age-related and regional distribution of the cohort as compared to the general population in Germany was provided considering the most recently available number of registered inhabitants by age groups and federal states from December 31^st^, 2022 according to the Federal Statistical Office of Germany (20).

## Results

**Figure 1:**
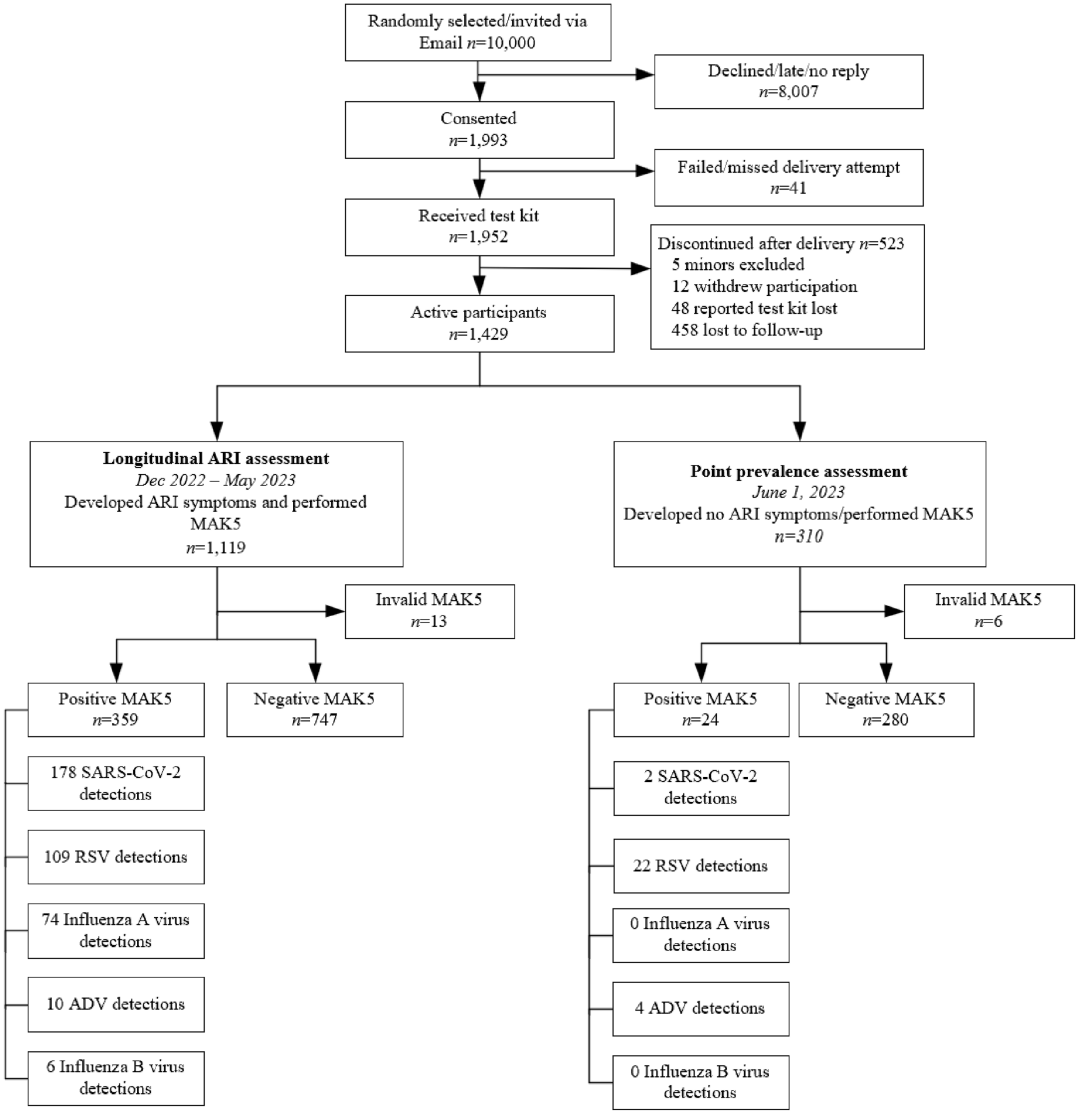
MAK5 participant flow diagram. ADV, adenovirus; RSV, respiratory syncytial virus; SARS-CoV-2, severe acute respiratory syndrome coronavirus 2; MAK5, multiple respiratory multipathogen antigen test kit.

Between December 7, 2022, and June 2, 2023, 1,429 (73.2%) of the 1,952 individuals who received a test kit performed self-testing (MAK5 participant flow diagram in Figure 1). Test reading accuracy was 100% comparing reported result and assay photography. Details on the study population, including demographics, underlying diseases and vaccination status are displayed in Supplementary Table 2. Compared to the general population in Germany, the distribution of participants per federal state was balanced except for overrepresentation of participants from North Rhine-Westphalia. Nationwide, only the age groups 60 to 80 years and above 80 years were underrepresented. Details on demographic and regional representativeness of the cohort are provided in Supplementary Tables 3, 4 and Supplementary Figure 2.

### Longitudinal ARI assessment, December 2022 – May 2023

**Figure 2:**
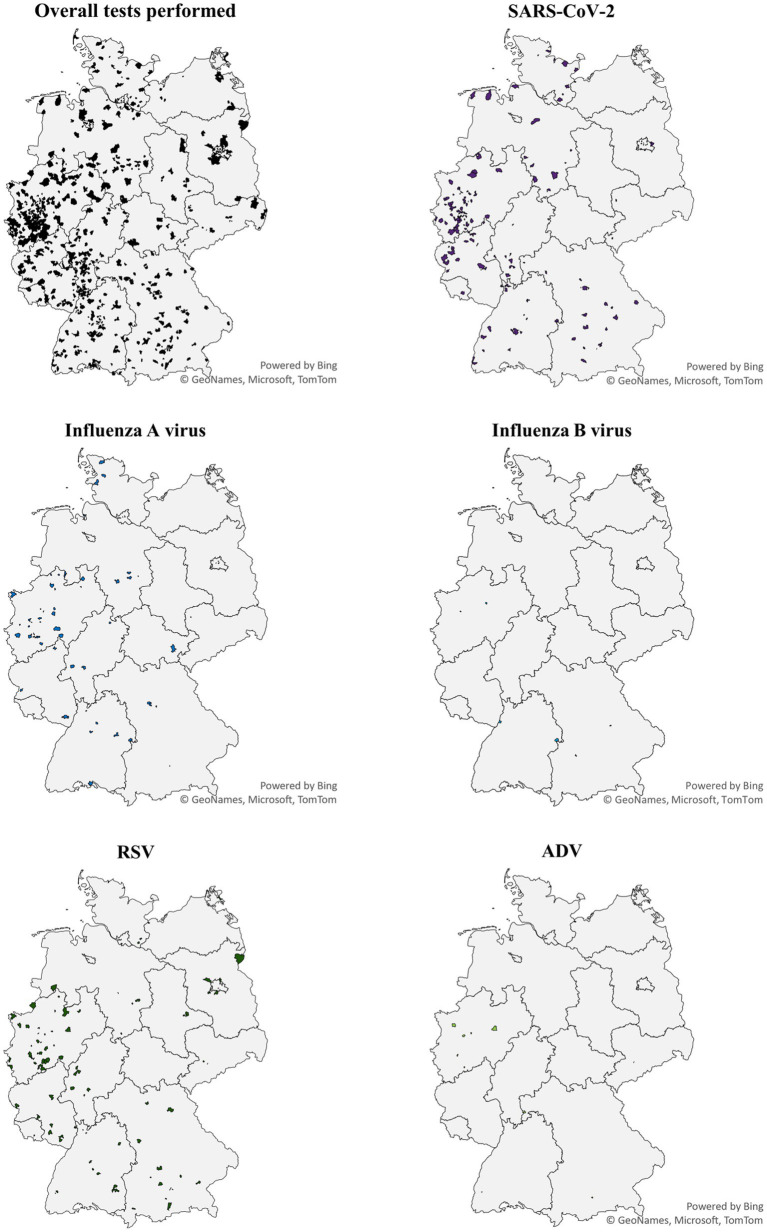
Geographical distribution of MAK5 test results December 2022–May 2023. ADV, adenovirus; RSV, respiratory syncytial virus; SARS-CoV-2, severe acute respiratory syndrome coronavirus 2.

ARI related symptoms were reported by 1,119 (57.3%) of the 1,952 participants who received the test kit (demographics given in Supplementary Table 2; geographical distribution in Figure 2).

Overall, 359 of 1,119 (32.1%) tests were positive with 17 (1.5%) co-detections, resulting in 377 detected pathogens overall. Thirteen tests (1.2%) were invalid or failed technically, and 747 (66.8%) were negative. The most frequently detected pathogen was SARS-CoV-2 (*n* = 178; 47.2%) followed by RSV (*n* = 109; 28.9%) and influenza A virus (*n* = 74; 19.6%). ADV was detected in 10 (2.7%) and influenza B virus in six (1.6%) samples only.

#### Incidences and co-detections

Incidences per calendar between the date of receipt of the test kit and May 31, 2023 are provided for each MAK5 pathogen separately alongside incidence values corrected for test sensitivity and number of active participants Supplementary Table 5.

The 17 MAK5 tests that detected more than one pathogen were positive for RSV (*n* = 14), influenza A (*n* = 8), ADV (*n* = 8), SARS-CoV-2 (*n* = 4), and influenza B virus (*n* = 1). RSV and ADV were co-detected in seven, and RSV and influenza A virus in five participants. In one participant influenza A and B virus, and SARS-CoV-2 were co-detected. Details are provided in Supplementary Table 6.

Demographic determinants of incidences including test results by age groups are given in Supplementary results and Supplementary Figures 2, 3.

#### Symptoms

Rhinitis was the most common symptom in participants who tested positive for ADV (*n* = 8; 80%), RSV (*n* = 86; 79.3%) and SARS-CoV-2 (*n* = 124; 69.7%).

Participants with influenza A virus detection had significantly more often cough (*n* = 61; 91.0%; *p* = 0,002), fever (*n* = 40; 59.7%; *p* < 0.001), conjunctivitis (*n* = 8; 11.9%; *p* = 0.002), and myalgia (*n* = 29; 43.1%; *p* = 0.001; Supplementary Table 7, Supplementary Table 8), as well as higher median number of symptoms (5 vs. 4; *p* < 0.001) compared to those with RSV or SARS-CoV-2. Headache (*n* = 33; 34.4%; *p* = 0.108) was less common in participants with RSV detection compared to those with influenza A virus and SARS-CoV-2 detection (Supplementary Figures 5–8).

In participants with negative MAK5 results, rhinitis was the most common symptom (*n* = 567; 75.9%).

Influenza B virus and ADV were excluded from these proportional comparisons due to small numbers. More details are given in the Supplementary results.

#### Medical history / underlying conditions

A total of 112 (62.9%) participants with SARS-CoV-2 detection reported prior laboratory-confirmed COVID-19, and 22 (12.3%) had received less than three vaccinations against COVID-19. Prior influenza vaccination in 22/23 was reported by 5 (83.3%) participants with influenza B virus and by 32 (43.2%) with influenza A virus detection. No significant differences were found in underlying chronic diseases.

#### Distribution of virus detections over time

The highest weekly number of influenza A virus detections was reported in mid-December (*n* = 30; 18.3%; AR 1.7%). The six influenza B virus detections were registered from January to April 2023.

RSV was the most frequently detected virus in the beginning of December (*n* = 25; 12.6%; AR 1.2%). After a second rise at the end of December (*n* = 15; 21.4; AR 1.0%), the number steadily decreased and plateaued until mid-May.

**Figure 3:**
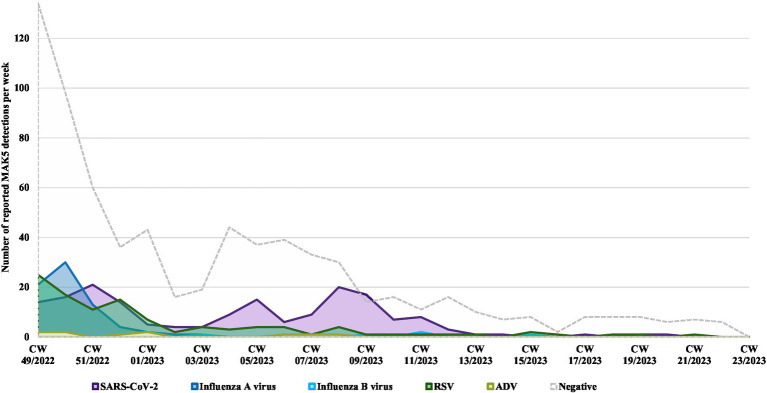
Reported MAK5 test results per calendar week (CW) December 2022–May 2023. ADV, adenovirus; RSV, respiratory syncytial virus; SARS-CoV-2, severe acute respiratory syndrome coronavirus; CW, calendar week.

The weekly number of reported SARS-CoV-2 detections fluctuated until end of March with highest number of detections at the end of December (*n* = 21; 20%; AR 1.3%), as well as the beginning (*n* = 15; 26.8%; AR 1.2%) and end of February (*n* = 20; 35.7%; AR 1.5%; Figure 3).

### Point prevalence assessment on June 01, 2023

Those 310 (22.1%) participants who had not developed ARI symptoms by May 31, 2023, performed the MAK5 test on June 1, 2023, irrespective of respiratory symptoms. Underlying cohort characteristics of single pathogen detections are displayed in Supplementary Table 2. Of these 310 participants, 258 (83.2%) reported to be asymptomatic for ARI on the day of testing. Participants who reported symptoms predominantly experienced mild symptoms, primarily rhinitis Supplementary Table 7.

Overall, 28 detections, including four RSV-ADV co-detections (1.3%) were reported from 24 (7.7%) positive participants. The most frequently detected virus was RSV (*n* = 22; 7.0%; AR 2.4%), followed by ADV (*n* = 4; 1.3%; AR 0.4%) and SARS-CoV-2 (*n* = 2; 0.6%; AR 0.2; Supplementary Figure 10). No influenza virus detections were reported. However, six invalid test results were assessed. No significant sex or age specific differences were found. Symptoms are reported in Supplementary results, Supplementary Table 8, Supplementary Figures 4–9.

## Discussion

The present citizen science study assessed the feasibility of self-testing for real-time detection of select ARI pathogens using multipathogen antigen rapid tests in the adult general population, regardless of whether participants sought medical care or not.

While influenza A virus and RSV were the most frequently detected pathogens in December 2022, SARS-CoV-2 was the most frequently detected pathogen over the entire longitudinal ARI assessment. Unexpectedly, we found a 7.7% RSV prevalence on June 1, 2023.

Seasonality and virus distribution patterns were mostly in line with reports from national institutions despite the absence of NAAT validation of MAK5 results (10). Differences were as follows. The Robert Koch-Institute reported consistently higher numbers of influenza (A and B) virus detections in those seeking medical attention compared to other respiratory pathogens including RSV and SARS-CoV-2 throughout December 2022, while in our study RSV was the most frequent pathogen in the second week of December (10). A likely explanation is that influenza causes more severe disease than RSV, prompting more patients to seek medical attention, thus being more visible in the Robert Koch-Institute monitoring data from medical practices and hospitals (21). Based on Germany’s adult population of 68.47 million inhabitants aged 20 years and above, our longitudinal data would at least translate into 10.89 million (15.9%) SARS-CoV-2-associated ARI between the beginning of December 2022 and end of May 2023 (20). Corresponding figures would be 6.64 million (9.7%) for RSV, and 4.52 million (6.6%) for influenza A virus (20). Considering there was only one test done per participant, our data may actually underestimate the true number of detections.

Point prevalence evaluation found a high rate of RSV, while local and national surveillance systems reported no RSV activity at all (10, 11). Almost two thirds of RSV positive participants were asymptomatic and the remaining had mostly mild rhinitis, unlikely seeking medical attention. ARI may have been confused with seasonal allergic rhinitis in spring, or even remained unnoticed due to low morbidity in otherwise healthy adults, especially during the summer season (22). This finding hints toward a hitherto unrecognized reservoir, but also questions the disease burden of RSV infection. Epidemiology of RSV in outpatients has not been investigated extensively, but RSV is a known major threat to immunocompromised patients, if not vaccinated (16, 23, 24). A community-based cohort study reported a significant impact of RSV infections in older adults also outside ARI-season (25). The PERCH study, although performed in children, has emphasized the challenges of distinguishing colonization from infection, or more particularly attributing causality to an organism detected. Of note, for RSV not only high overall detection rates were reported in that study, but also uncommon detection in controls, therefore proving high predictive value for etiological attribution (26). On the other hand, this argument may specifically apply for NAAT due to its high sensitivity (18). On the contrary, for SARS-CoV-2, it is well described that even asymptomatic individuals are contagious highlighting the valuable contribution of self-testing to surveillance efforts in a pandemic (27).

The findings of our study need to be interpreted in the context of certain limitations. Rapid antigen testing is less sensitive and specific than NAAT while validation results from the MAK5 manufacturer show comparably high sensitivity for MAK5. Self-testing may be prone to inferior test performance, although reliable results have been documented (28–30). MAK5 does not comprise some presumably highly prevalent viruses, such as rhinovirus, that are consequently not captured. Another explanation for the high proportion of negative test results immediately after test kit shipment may be a reduced viral load due to delayed testing of an already ongoing ARI. Only the first symptomatic episode per participant was analyzed, while the cohort decreased in numbers. Consequently, calculated incidences may be weighted differently depending on the timing to account for reduced statistical power toward the end of the study period. Accordingly, this feasibility study only allowed for the comparison of dynamics of circulating pathogens and timing of waves, rather than precise incidence numbers.

## Conclusion

MAK5 self-testing qualifies as a useful tool to describe ARI incidence in individuals not seeking medical attention. In the context of pandemic preparedness, individuals from the general population who self-test at regular intervals could serve as a sentinel cohort for real-time monitoring of epidemiology. Our study successfully identified a segment of the population that, while not seeking medical care, represents a potential reservoir for ARI pathogens. This highlights the feasibility and value of gathering relevant epidemiological data through a citizen science approach.

## Data Availability

The raw data supporting the conclusions of this article will be made available by the authors, without undue reservation.

## References

[1] MontoAS. Epidemiology of viral respiratory infections. Am J Med. (2002) 112:4–12. doi: 10.1016/S0002-9343(01)01058-0, PMID: 11955454

[2] Dakhama A Lee YM Gelfand EW. Virus-induced airway dysfunction: pathogenesis and biomechanisms. Pediatr Infect Dis J. (2005) 24:S159–69. doi: 10.1097/01.inf.0000188155.46381.15, PMID: 16378041

[3] Aoki FY Macleod MD Paggiaro P Carewicz O El Sawy A Wat C. Early administration of oral oseltamivir increases the benefits of influenza treatment. J Antimicrob Chemother. (2003) 51:123–9. doi: 10.1093/jac/dkg007, PMID: 12493796

[4] Wilson P Zumla A. Transmission and prevention of acute viral respiratory tract infections in hospitals. Curr Opin Pulm Med. (2019) 25:220–4. doi: 10.1097/mcp.0000000000000566, PMID: 30730312

[5] Zhang N Wang L Deng X Liang R Su M He C. Recent advances in the detection of respiratory virus infection in humans. J Med Virol. (2020) 92:408–17. doi: 10.1002/jmv.25674, PMID: 31944312 PMC7166954

[6] Klompas M Imrey PB Yu PC Rhee C Deshpande A Haessler S. Respiratory viral testing and antibacterial treatment in patients hospitalized with community-acquired pneumonia. Infect Control Hosp Epidemiol. (2021) 42:817–25. doi: 10.1017/ice.2020.1312, PMID: 33256870 PMC8665830

[7] Ostrow O Savlov D Richardson SE Friedman JN. Reducing unnecessary respiratory viral testing to promote high-value care. Pediatrics. (2022) 149:e2020042366. doi: 10.1542/peds.2020-042366, PMID: 35102418

[8] Rigoine de Fougerolles T Puig-Barbera J Kassianos G Vanhems P Schelling J Crepey P. A comparison of coronavirus disease 2019 and seasonal influenza surveillance in five European countries: France, Germany, Italy, Spain and the United Kingdom. Influenza Other Respir Viruses. (2022) 16:417–28. doi: 10.1111/irv.12941, PMID: 34866344 PMC8983920

[9] Byington CL Ampofo K Stockmann C Adler FR Herbener A Miller T. Community surveillance of respiratory viruses among families in the Utah better identification of germs-longitudinal viral epidemiology (BIG-LoVE) study. Clin Infect Dis. (2015) 61:1217–24. doi: 10.1093/cid/civ486, PMID: 26245665 PMC4583580

[10] Arbeitsgemeinschaft Influenza. Robert Koch-Institut. (2023). Available online at: https://influenza.rki.de/Wochenberichte/2022_2023/2023-28.pdf (Accessed 24.07.2023).

[11] GrippeWeb. Robert Koch-Institut, (2023). Available online at: https://edoc.rki.de/bitstream/handle/176904/11335/Wochenbericht_GrippeWeb_2023KW44.pdf?sequence=1&isAllowed=y (Accessed 30.12.2023).

[12] Bayer C Remschmidt C An Der Heiden M Tolksdorf K Herzhoff M Kaersten S. Internet-based syndromic monitoring of acute respiratory illness in the general population of Germany, weeks 35/2011 to 34/2012. Euro Surveill. (2014) 19:20684. doi: 10.2807/1560-7917.ES2014.19.4.20684, PMID: 24507468

[13] van Noort SP Muehlen M Rebelo de Andrade H Koppeschaar C Lima Lourenço JM Gomes MG. Gripenet: an internet-based system to monitor influenza-like illness uniformly across Europe. Euro Surveill. (2007) 12:5–6. doi: 10.2807/esm.12.07.00722-en, PMID: 17991409

[14] Paolotti D Carnahan A Colizza V Eames K Edmunds J Gomes G. Web-based participatory surveillance of infectious diseases: the Influenzanet participatory surveillance experience. Clin Microbiol Infect. (2014) 20:17–21. doi: 10.1111/1469-0691.12477, PMID: 24350723 PMC7128292

[15] Haussig JM Targosz A Engelhart S Herzhoff M Prahm K Buda S. Feasibility study for the use of self-collected nasal swabs to identify pathogens among participants of a population-based surveillance system for acute respiratory infections (GrippeWeb-plus)—Germany, 2016. Influenza Other Respir Viruses. (2019) 13:319–30. doi: 10.1111/irv.12644, PMID: 30925029 PMC6586186

[16] Shi T Denouel A Tietjen AK Campbell I Moran E Li X. Global disease burden estimates of respiratory syncytial virus-associated acute respiratory infection in older adults in 2015: a systematic review and Meta-analysis. J Infect Dis. (2020) 222:S577–83. doi: 10.1093/infdis/jiz059, PMID: 30880339

[17] Shidlovskaya EV Kuznetsova NA Divisenko EV Nikiforova MA Siniavin AE Ogarkova DA. The value of rapid antigen tests for identifying carriers of viable SARS-CoV-2. Viruses. (2021) 13:2012. doi: 10.3390/v13102012, PMID: 34696442 PMC8537476

[18] Eyre DW Futschik M Tunkel S Wei J Cole-Hamilton J Saquib R. Performance of antigen lateral flow devices in the UK during the alpha, delta, and omicron waves of the SARS-CoV-2 pandemic: a diagnostic and observational study. Lancet Infect Dis. (2023) 23:922–32. doi: 10.1016/s1473-3099(23)00129-9, PMID: 37001541 PMC10048397

[19] Salmanton-García J Stewart FA Heringer S Koniordou M Álvarez-Barco E Argyropoulos CD. VACCELERATE volunteer registry: a European study participant database to facilitate clinical trial enrolment. Vaccine. (2022) 40:4090–7. doi: 10.1016/j.vaccine.2022.05.022, PMID: 35659449 PMC9159788

[20] Statistisches-Bundesamt. *Bevölkerung - Zahl der Einwohner in Deutschland nach relevanten Altersgruppen am 31. Dezember 2022 (in Millionen)*. (2023). Available at: https://de.statista.com/statistik/daten/studie/1365/umfrage/bevoelkerung-deutschlands-nach-altersgruppen/ (Accessed August 03, 2023).

[21] VanWormer JJ Sundaram ME Meece JK Belongia EA. A cross-sectional analysis of symptom severity in adults with influenza and other acute respiratory illness in the outpatient setting. BMC Infect Dis. (2014) 14:231. doi: 10.1186/1471-2334-14-231, PMID: 24884932 PMC4013802

[22] Bauchau V Durham SR. Prevalence and rate of diagnosis of allergic rhinitis in Europe. Eur Respir J. (2004) 24:758–64. doi: 10.1183/09031936.04.00013904, PMID: 15516669

[23] Falsey AR Hennessey PA Formica MA Cox C Walsh EE. Respiratory syncytial virus infection in elderly and high-risk adults. N Engl J Med. (2005) 352:1749–59. doi: 10.1056/NEJMoa043951, PMID: 15858184

[24] Addo M Cornely O Denkinger M Ertl G Herold S Pletz M. RSV vaccination strategies for high-risk patients 2023: a collaborative position paper by leading German medical societies and organizations. Infection. (2024) 52:285–8. doi: 10.1007/s15010-023-02141-5, PMID: 38060068 PMC10811136

[25] Juhn YJ Wi CI Takahashi PY Ryu E King KS Hickman JA. Incidence of respiratory syncytial virus infection in older adults before and during the COVID-19 pandemic. JAMA Netw Open. (2023) 6:e2250634. doi: 10.1001/jamanetworkopen.2022.50634, PMID: 36662530 PMC9860520

[26] O’Brien KL Baggett HC Brooks WA Feikin DR Hammitt LL Higdon MM. Causes of severe pneumonia requiring hospital admission in children without HIV infection from Africa and Asia: the PERCH multi-country case-control study. Lancet. (2019) 394:757–79. doi: 10.1016/s0140-6736(19)30721-4, PMID: 31257127 PMC6727070

[27] Tan F Wang K Liu J Liu D Luo J Zhou R. Viral transmission and clinical features in asymptomatic carriers of SARS-CoV-2 in Wuhan, China. Front Med (Lausanne). (2020) 7:547. doi: 10.3389/fmed.2020.00547, PMID: 33015099 PMC7461982

[28] Akmatov MK Gatzemeier A Schughart K Pessler F. Equivalence of self- and staff-collected nasal swabs for the detection of viral respiratory pathogens. PLoS One. (2012) 7:e48508. doi: 10.1371/journal.pone.0048508, PMID: 23155387 PMC3498275

[29] Stemler J Salmanton-García J Weise B Többen C Joisten C Fleig J. A pilot surveillance report of SARS-CoV-2 rapid antigen test results among volunteers in Germany, 1st week of July 2022. Infection. (2023) 51:465–9. doi: 10.1007/s15010-022-01931-7, PMID: 36279033 PMC9590393

[30] Plymoth A Rotzén-Östlund M Zweygberg-Wirgart B Sundin CG Ploner A Nyrén O. Self-sampling for analysis of respiratory viruses in a large-scale epidemiological study in Sweden. Euro Surveill. (2015) 20:21063. doi: 10.2807/1560-7917.ES2015.20.11.21063, PMID: 25811646

