# Supplementary material for: Self-testing for 5 respiratory viruses in adult VACCELERATE volunteers in Germany—a pilot study on multi-pathogen rapid antigen testing to monitor community-acquired acute respiratory infections
Source: Front Public Health. 2025 Sep 3;13:1638280. doi: 10.3389/fpubh.2025.1638280 (PMC12440861; doi:10.3389/fpubh.2025.1638280)
Supplement: Supplementary file 1 [file Supplementary_file_1.docx]

**Supplementary Material**

[1 Supplementary Results 2](#_Toc206369405)

[Longitudinal ARI assessment (December 2022 – May, 2023) 2](#_Toc206369406)

[Study population 2](#_Toc206369407)

[Demographics 2](#_Toc206369408)

[Symptoms 2](#_Toc206369409)

[Point prevalence assessment (June 1, 2023) 3](#_Toc206369410)

[Symptoms 3](#_Toc206369411)

[2 Supplementary Tables 4](#_Toc206369412)

[Supplementary Table 1. 4](#_Toc206369413)

[Supplementary Table 2. 5](#_Toc206369414)

[Supplementary Table 3. 7](#_Toc206369415)

[Supplementary Table 4*.* 8](#_Toc206369416)

[Supplementary Table 5. 9](#_Toc206369417)

[Supplementary Table 6*.* 10](#_Toc206369418)

[Supplementary Table 7. 11](#_Toc206369419)

[Supplementary Table 8*.* 12](#_Toc206369420)

[Supplementary Figure 1. 13](#_Toc206369421)

[Supplementary Figure 2. 14](#_Toc206369422)

[Supplementary Figure 3. 15](#_Toc206369423)

[Supplementary Figure 4. 16](#_Toc206369424)

[Supplementary Figure 5. 17](#_Toc206369425)

[Supplementary Figure 6. 18](#_Toc206369426)

[Supplementary Figure 7. 19](#_Toc206369427)

[Supplementary Figure 8. 20](#_Toc206369428)

[Supplementary Figure 9. 21](#_Toc206369429)

[Supplementary Figure 10. 22](#_Toc206369430)

# Supplementary Results

## Longitudinal ARI assessment (December 2022 – May, 2023)

## Study population

While 473 (23.7%) volunteers never replied after shipment of the test kit, 48 (2.4%) participants reported on the loss of the MAK5 test kit, 41 (2.1%) individuals replied that they had not received a test kit and 12 (0.6%) volunteers withdrew from study participation, overall resulting in a drop-out rate of 28.8%. A total of 13 (0.7%) participants reported a test result not of themselves but from somebody else (e.g., child, spouse). Of these, five gave it to children of minor age, who were excluded from this analysis, and seven to children of adult age. The adolescent individuals tested instead were subsequently invited to register in the Volunteer Registry to participate in the present study.

## Demographics

While no significant gender specific differences were found in terms of MAK5 pathogen detections, we observed age related differences (p<0.001). Influenza B virus and ADV were excluded from the proportional comparison due to comparatively low numbers of infections. Influenza A virus infection was most frequently observed in participants between 30 and 39 years (*n*=35, 47.3%), whereas SARS-CoV-2 infection was similarly represented in the age groups 30-39 (*n*=38, 21.3%), 40-49 (n=41, 23.0%), 50-59 (*n*=42, 23.6%) and 60-69 (*n*=36, 20.2%). Similarly, RSV was equally represented in multiple age groups, with 30-39 (*n*=34, 31.2%), 40-49 (*n*=28, 25.7%), 50-59 (*n*=24, 22.0%), overall resulting in lower mean age compared to SARS-CoV-2 (Table 2, Supplementary figures 2 and 3).

## Symptoms

Pharyngitis occurred in 578 (62.9%) participants ≤60 years of age and in 83 (34.2%) participants ≥70 years of age (p<0.001). Fever was less common in participants ≥70 years (n=6; 15.8%) compared to all other age groups, while observed in 73 (25.9%) participants between 40 and 49 years (p=n.s.). Conjunctivitis was reported in 13.2% of those ≥70 years of age, in 11.6% of participants between 50 and 59 years, and in 5.6% of participants <50 years (range 4.6%-6.4%; p=0.034).

## Point prevalence assessment (June 1, 2023)

## Symptoms

A total of eight (36.4%) participants, who tested positive for RSV reported ARI-related symptoms. Most commonly reported symptoms were rhinitis (*n*=5, 22.7%), pharyngitis (*n*=3, 13.6%) and cough (*n*=2, 9.1%). Regarding ADV, 75% (*n*=3) were asymptomatic, whereas one participant had tonsilitis and sinus pressure. Both participants with SARS-CoV-2 infection reported on rhinitis. Further reported symptoms were fever, pharyngitis, myalgia and headache (*n*=1, each).

# Supplementary Tables

Supplementary Table 1. **Clinical performance of the multipathogen antigen test kit (MAK5) (BioTeke Corporation (Wuxi) Co., Ltd., Wuxi, Jiangsu, China) ^24^**

|  | Sensitivity  (95% CI) | Specificity  (95% CI) | Coincidence rate  (95% CI) | Kappa-value |
| --- | --- | --- | --- | --- |
| SARS-CoV-2 | 96.36  (90.95; 99.00) | 100  (99.19; 100.00) | 99.29  (98.20; 99.81) | 0.9771 |
| Influenza A | 94.96  (89.35; 98.13) | 100  (99.18; 100) | 98.95  (97.71; 99.61) | 0.9675 |
| Influenza B | 95.37  (89.53; 98.48) | 100  (99.18; 100) | 99.10  (97.91; 99.71) | 0.9707 |
| RSV A/B | 93.81  (87.65; 97.47) | 100  (99.19; 100) | 98.77  (97.47; 99.50) | 0.9604 |
| Adenovirus | 94.29  (87.98; 97.87) | 100  (99.19; 100) | 98.93  (97.68; 99.61) | 0.9640 |

*Evaluated with clinical samples by comparison with test results from the same samples using light cycler PCR (Roche) and Cobas Z480 Analyzer (Roche).CI Confidence Interval*

Supplementary Table 2. **Distribution of single viral detections* by cohort demographics, vaccination history and medical conditions**

|  | **Negative test result** | | **SARS-CoV-2** | | **Influenza A virus** | | **Influenza B virus** | | **RSV** | | **ADV** | | **Total**** | |  | **Negative test result** | | **SARS-CoV-2** | | **RSV** | | **ADV** | | **Total**** | |
| --- | --- | --- | --- | --- | --- | --- | --- | --- | --- | --- | --- | --- | --- | --- | --- | --- | --- | --- | --- | --- | --- | --- | --- | --- | --- |
|  | **Longitudinal assessment: December 2022 – May 2023** | | | | | | | | | | | | | |  | **Point prevalence assessment on June 01, 2023** | | | | | | | | | |
|  | **n [%]** | | | | | | | | | | | | | |  | **n [%]** | | | | | | | | | |
| **Sex** |  |  |  |  |  |  |  |  |  |  |  |  |  |  |  |  |  |  |  |  |  |  |  |  |  |
| Female | 458 | 61.3 | 117 | 65.7 | 43 | 58.1 | 2 | 33.3 | 71 | 65.1 | 9 | 90.0 | 706 | 62.1 |  | 172 | 61.2 | 2 | 100.0 | 14 | 63.6 | 1 | 25.0 | 191 | 60.8 |
| Male | 288 | 38.6 | 61 | 34.3 | 31 | 41.9 | 4 | 66.7 | 38 | 34.0 | 1 | 10.0 | 430 | 37.8 |  | 107 | 38.1 | - | - | 8 | 36.4 | 3 | 75.0 | 121 | 38.5 |
| Non-binary† | 1 | 0.1 | - | - | - | - | - | - | - | - | - | - | 1 | 0.1 |  | 2 | 0.7 | - | - | - | - | - | - | 2 | 0.6 |
| **Age group [yrs]** Median (IQR) [range] | 48 (38-57)  [20-88] | | 49 (37-58)  [21-76] | | 38 (33-46)  [7-56] | | 39.5 (31-45)  [27-49] | | 42 (33-52)  [4-71] | | 39 (32-49)  [3-56] | | 46 (38-57)  [3-88] | |  | 51 (38-58)  [20-91] | | 60.5 (52-69)  [52-69] | | 56.5 (45-60)  [34-72] | | 45.5 (43.5-59)  [42-72] | | 48 (37-58)  [20-91] | |
| 18-29 | 63 | 8.4 | 15 | 8.4 | 9 | 12.2 | 1 | 16.7 | 13 | 11.9 | - | - | 102 | 9.0 |  | 15 | 5.3 | - | - | - | - | - | - | 15 | 4.8 |
| 30-39 | 175 | 23.4 | 38 | 21.3 | 35 | 47.3 | 2 | 33.3 | 34 | 31.2 | 5 | 50.0 | 291 | 25.6 |  | 42 | 14.9 | - | - | 2 | 9.1 | - | - | 44 | 14.0 |
| 40-49 | 200 | 27.2 | 41 | 23.0 | 14 | 18.9 | 3 | 50.0 | 28 | 25.7 | 3 | 30.0 | 290 | 25.5 |  | 58 | 20.6 | - | - | 7 | 31.8 | 3 | 75.0 | 68 | 21.7 |
| 50-59 | 177 | 23.7 | 42 | 23.6 | 15 | 20.3 | - | - | 24 | 22.0 | 2 | 20.0 | 265 | 23.3 |  | 87 | 31.0 | 1 | 50.0 | 7 | 31.8 | - | - | 95 | 30.6 |
| 60-69 | 99 | 13.3 | 36 | 20.2 | - | - | - | - | 9 | 8.3 | - | - | 148 | 13.0 |  | 59 | 21.0 | 1 | 50.0 | 4 | 18.2 | - | - | 65 | 20.7 |
| 70+ | 30 | 4 | 6 | 3.4 | - | - | - | - | 1 | 0.9 | - | - | 37 | 3.3 |  | 20 | 7.1 | - | - | 2 | 9.1 | 1 | 25.0 | 26 | 8.3 |
| **Prior COVID-19 vaccination** Median (IQR) [range] | 3 (3-4)  [0-6] | | 4 (3-4)  [0-6] | | 3 (3-4)  [0-6] | | 3 (3-4)  [3-4] | | 3 (3-4)  [0-5] | | 3 (3-4)  [3-5] | | 3 (3-4)  [0-6] | |  | 3 (3-4)  [0-6] | | 3.5 (3-4)  3-4] | | 4 (3-4)  [3-5] | | 4 (3-4)  [3-4] | | 3 (3-4)  [0-6] | |
| 0 | 32 | 4.5 | 11 | 6.2 | 4 | 5.4 | - | - | 4 | 3.7 | - | - | 52 | 4.6 |  | - | - | - | - | - | - | - | - | - | - |
| 1 | 13 | 1.7 | 2 | 1.1 | 1 | 1.4 | - | - | 2 | 1.8 | - | - | 19 | 1.7 |  | - | - | - | - | - | - | - | - | - | - |
| 2 | 44 | 5.9 | 9 | 5.1 | 4 | 5.4 | - | - | 8 | 7.3 | - | - | 65 | 5.7 |  | - | - | - | - | - | - | - | - | - | - |
| 3 | 278 | 37.2 | 51 | 28.7 | 31 | 41.9 | 3 | 50.0 | 47 | 43.1 | 6 | 60.0 | 420 | 36.9 |  | - | - | - | - | - | - | - | - | - | - |
| 4 | 233 | 31.2 | 65 | 36.5 | 14 | 18.9 | 2 | 33.3 | 30 | 27.5 | 3 | 30.0 | 351 | 30.9 |  | - | - | - | - | - | - | - | - | - | - |
| 5 | 50 | 6.7 | 17 | 9.60 | 9 | 12.2 | - | - | 7 | 6.4 | 1 | 10.0 | 85 | 7.5 |  | - | - | - | - | - | - | - | - | - | - |
| 6 | 11 | 1.5 | 3 | 1.7 | 1 | 1.4 | - | - | - | - | - | - | 16 | 1.4 |  | - | - | - | - | - | - | - | - | - | - |
| **Prior COVID-19 infection** | 450 | 62.2 | 112 | 62.9 | 44 | 59.5 | 5 | 83.3 | 69 | 63.3 | 9 | 90.0 | 695 | 61.1 |  | 159 | 56.6 | 2 | 100.0 | 12 | 54.5 | 2 | 50.0 | 177 | 56.4 |
| **Influenza VACC 22/23** | 356 | 47.7 | 90 | 50.6 | 32 | 43.2 | 5 | 83.3 | 42 | 38.5 | 4 | 40.0 | 536 | 47.1 |  | 157 | 55.9 | 1 | 50.0 | 12 | 54.5 | 2 | 50.0 | 175 | 55.7 |
| **Underlying conditions** |  |  |  |  |  |  |  |  |  |  |  |  |  |  |  |  |  |  |  |  |  |  |  |  |  |
| Hypertension | 98 | 13.1 | 25 | 14.0 | 6 | 8.1 | 1 | 16.7 | 14 | 12.8 | - | - | 147 | 12.9 |  | 55 | 19.6 | 1 | 50.0 | 2 | 9.1 | - | - | 60 | 19.1 |
| Coronary heart disease | 8 | 1.1 | 2 | 1.1 | - | - | - | - | 2 | 1.8 | - | - | 12 | 1.1 |  | 6 | 2.1 | - | - | 1 | 4.5 | 1 | 25.0 | 9 | 2.9 |
| Heart failure | 6 | 0.8 | 1 | 0.6 | - | - | - | - | 1 | 0.9 | - | - | 8 | 0.7 |  | 1 | 0.4 | - | - | 1 | 4.5 | - | - | 2 | 0.6 |
| Asthma/COPD | 73 | 9.8 | 18 | 10.1 | 3 | 4.1 | 1 | 16.7 | 11 | 10.1 | - | - | 107 | 9.4 |  | 32 | 11.4 | - | - | 3 | 13.6 | - | - | 36 | 11.5 |
| Chronic kidney disease | 8 | 1.1 | - | - | - | - | - | - | 2 | 1.8 | - | - | 11 | 1.0 |  | 1 | 0.4 | - | - | 1 | 4.5 | - | - | 2 | 0.6 |
| ≥20 kg overweight | 70 | 9.4 | 17 | 9.6 | 7 | 9.5 | 1 | 16.7 | 9 | 8.3 | - | - | 104 | 9.1 |  | 29 | 10.3 | - | - | 3 | 13.6 | 2 | 50.0 | 35 | 11.1 |
| Diabetes mellitus | 24 | 3.2 | 4 | 2.2 | 1 | 1.4 | - | - | - | - | - | - | 30 | 2.6 |  | 11 | 3.9 | 1 | 50.0 | - | - | - | - | 13 | 4.1 |
| HIV | 6 | 0.8 | 4 | 2.2 | 1 | 1.4 | - | - | 1 | 0.9 | - | - | 13 | 1.1 |  | 3 | 1.1 | - | - | 1 | 4.5 | 1 | 25.0 | 5 | 1.6 |
| Active cancer (≥2 yrs) | 15 | 2.0 | 1 | 0.6 | - | - | - | - | 1 | 0.9 | - | - | 17 | 1.5 |  | 4 | 1.4 | - | - | 1 | 4.5 | - | - | 5 | 1.6 |
| Chronic GIT disease | 7 | 0.9 | 3 | 1.7 | - | - | - | - | 3 | 2.8 | - | - | 14 | 1.2 |  | 2 | 0.7 | - | - | - | - | - | - | 2 | 0.6 |
| History of stroke | 8 | 1.1 | - | - | - | - | - | - | 1 | 0.9 | - | - | 10 | 0.9 |  | - | - | - | - | 1 | 4.5 | - | - | 1 | 0.3 |

*ADV, adenovirus; COPD, chronic obstructive pulmonary disease; COVID-19, coronavirus disease 2019; GIT, gastrointestinal; HIV, human immunodeficiency virus; INF, infection; IQR, interquartile range; RSV, respiratory syncytial virus; SARS-CoV-2, severe acute respiratory syndrome coronavirus 2; yrs, years; VACC, vaccination*

* Co-detections separately listed (test results > participants)

** Including invalid test results

† Corresponds to legal term “divers” in German language

Supplementary Table 3. **Geographical distribution of participants in Germany by federal states as compared to the general population in Germany**

| State | State code | Inhabitants  N [%] | Participants  N [%] | Difference of absolute percentage points [%] |
| --- | --- | --- | --- | --- |
| Baden-Württemberg | BW | 11,148,904 [13.4] | 211 [10.6] | -2.8 |
| Bavaria | BY | 13,203,592 [15.8] | 237 [11.9] | -3.9 |
| Berlin | BE | 3,689,708 [4.4] | 99 [5.0] | 0.6 |
| Brandenburg | BB | 2,546,685 [3.1] | 30 [1.5] | -1.5 |
| Bremen | HB | 663,567 [0.8] | 16 [0.8] | 0.0 |
| Hamburg | HH | 1,904,212 [2.3] | 39 [2.0] | -0.3 |
| Hesse | HE | 6,313,614 [7.6] | 162 [8.1] | 0.6 |
| Lower Saxony | NI | 8,045,829 [9.6] | 120 [6.0] | -3.6 |
| Mecklenburg-Vorpommern | MV | 1,605,259 [1.9] | 14 [0.7] | -1.2 |
| North Rhine-Westphalia | NW | 17,944,923 [21.5] | 820 [41.1] | 19.7 |
| Rhineland-Palatinate | RP | 4,126,872 [4.9] | 89 [4.5] | -0.5 |
| Saarland | SL | 1,005,796 [1.2] | 16 [0.8] | -0.4 |
| Saxony | SN | 4,036,369 [4.8] | 42 [2.1] | -2.7 |
| Saxony-Anhalt | ST | 2,155,742 [2.6] | 20 [1.0] | -1.6 |
| Schleswig-Holstein | SH | 2,936,486 [3.5] | 52 [2.6] | -0.9 |
| Thuringia | TH | 2,099,527 [2.5] | 22 [1.1] | -1.4 |
| Not classified | - | - | 6 [0.3] | - |
| Total | - | 83,427,085 [100.0] | 1,995 [100.0] | 0.0 |

**Federal Statistical Office of Germany. Number of inhabitants in Germany by federal state on December 31, 2022.*

Supplementary Table 4. **Distribution of participants by age groups compared to the general population in Germany**

| *Age* [years] | Inhabitants  N [%] | Participants  N [%] | Difference of absolute percentage points [%] |
| --- | --- | --- | --- |
| Overall | 84,358,845 [100.0] | 1993 [100.0] | 0.0 |
| 20 - 40 | 20,636,488 [24.5] | 682 [34.2] | 9.7 |
| 40 - 60 | 22,999,053 [27.3] | 971 [48.7] | 21.4 |
| 60 - 80 | 18,749,279 [22.2] | 328 [16.4] | -5.8 |
| > 80 | 6,088,104 [7.2] | 9 [0.5] | -6.8 |
| Not classified | - | 5 [0.3] | - |

**Federal Statistical Office of Germany. Number of inhabitants in Germany by relevant age group on December 31, 2023.*

Supplementary Table 5. Incidences per calendar week (CW) including attack rates (AR) December 2022 – May 2023

|  | **Negative** | | **SARS-CoV-2** | | | **Influenza A virus** | | | **Influenza B virus** | | | **RSV** | | | **ADV** | | | **Invalid** | | **Total** | | **Active participants** | |
| --- | --- | --- | --- | --- | --- | --- | --- | --- | --- | --- | --- | --- | --- | --- | --- | --- | --- | --- | --- | --- | --- | --- | --- |
| **Calendar week** | **n** | **%** | **n** | **%** | **Incidence [%] (*)** | **n** | **%** | **Incidence [%] (*)** | **n** | **%** | **Incidence [%] (*)** | **n** | **%** | **Incidence [%] (*)** | **n** | **%** | **Incidence [%] (*)** | **n** | **%** | **n** | **%** | **n** |  |
| 2022 49 | 134 | 67.3 | 14 | 7.0 | 1.0 | 21 | 10.6 | 1.5 | - | - | - | 25 | 12.6 | 1.7 (1.9) | 2 | 1.0 | 0.1 | 3 | 1.5 | 199 | 100.0 | 1429 |  |
| 2022 50 | 98 | 59.8 | 16 | 9.8 | 1.3 | 30 | 18.3 | 2.4 (2.6) | - | - | - | 17 | 10.4 | 1.4 (1.5) | 2 | 1.2 | 0.2 | 1 | 0.6 | 164 | 100.0 | 1233 |  |
| 2022 51 | 60 | 57.1 | 21 | 20.0 | 2.0 | 13 | 12.4 | 1.2 (1.3) | - | - | - | 11 | 10.5 | 1.0 (1.1) | - | - | - | - | - | 105 | 100.0 | 1074 |  |
| 2022 52 | 36 | 51.4 | 14 | 20.0 | 1.4 (1.5) | 4 | 5.7 | 0.4 | - | - | - | 15 | 21.4 | 1.5 (1.7) | 1 | 1.4 | 0.1 | - | - | 70 | 100.0 | 969 |  |
| 2023 01 | 43 | 71.7 | 5 | 8.30 | 0.6 | 2 | 3.3 | 0.2 | 1.0 | 1.7 | 0.1 | 7 | 11.7 | 0.8 | 2 | 3.3 | 0.2 | - | - | 60 | 100.0 | 902 |  |
| 2023 02 | 16 | 66.7 | 4 | 16.7 | 0.5 | 1 | 4.2 | 0.1 | - | - | - | 2 | 8.3 | 0.2 (0.3) | - | - | - | 1 | 4.2 | 24 | 100.0 | 846 |  |
| 2023 03 | 19 | 61.3 | 4 | 12.9 | 0.5 | 1 | 3.2 | 0.1 | 1.0 | 3.2 | 0.1 | 4 | 12.9 | 0.5 | - | - | - | 2 | 6.5 | 31 | 100.0 | 822 |  |
| 2023 04 | 44 | 78.6 | 9 | 16.1 | 1.1 (1.2) | - | - | - | - | - | - | 3 | 5.4 | 0.4 | - | - | - | - | - | 56 | 100.0 | 791 |  |
| 2023 05 | 37 | 66.1 | 15 | 26.8 | 2.0 (2.1) | - | - | - | - | - | - | 4 | 7.1 | 0.5 (0.6) | - | - | - | - | - | 56 | 100.0 | 735 |  |
| 2023 06 | 39 | 76.5 | 6 | 11.8 | 0.9 | - | - | - | - | - | - | 4 | 7.8 | 0.6 | 1 | 2.0 | 0.1 (0.2) | 1 | 2.0 | 51 | 100.0 | 679 |  |
| 2023 07 | 33 | 75 | 9 | 20.5 | 1.4 (1.5) | - | - | - | - | - | - | 1 | 2.3 | 0.2 | 1 | 2.3 | 0.2 | - | - | 44 | 100.0 | 628 |  |
| 2023 08 | 30 | 53.6 | 20 | 35.7 | 3.4 (3.5) | - | - | - | - | - | - | 4 | 7.1 | 0.7 | 1 | 1.8 | 0.2 | 1 | 1.8 | 56 | 100.0 | 585 |  |
| 2023 09 | 14 | 41.2 | 17 | 50.0 | 3.2 (3.3) | 1 | 2.9 | 0.2 | 1.0 | 2.9 | 0.2 | 1 | 2.9 | 0.2 | - | - | - | - | - | 34 | 100.0 | 530 |  |
| 2023 10 | 16 | 64 | 7 | 28.0 | 1.4 (1.5) | - | - | - | - | - | - | 1 | 4.0 | 0.2 | - | - | - | 1 | 4.0 | 25 | 100.0 | 497 |  |
| 2023 11 | 11 | 47.8 | 8 | 34.8 | 1.7 (1.8) | - | - | - | 2.0 | 8.7 | 0.4 (0.5) | 1 | 4.3 | 0.2 | - | - | - | 1 | 4.3 | 23 | 100.0 | 472 |  |
| 2023 12 | 16 | 80 | 3 | 15.0 | 0.7 | - | - | - | - | - | - | 1 | 5.0 | 0.2 | - | - | - | - | - | 20 | 100.0 | 449 |  |
| 2023 13 | 10 | 83.3 | 1 | 8.3 | 0.2 | - | - | - | - | - | - | 1 | 8.3 | 0.2 | - | - | - | - | - | 12 | 100.0 | 429 |  |
| 2023 14 | 7 | 87.5 | 1 | 12.5 | 0.2 | - | - | - | - | - | - | - | - | - | - | - | - | - | - | 8 | 100.0 | 417 |  |
| 2023 15 | 8 | 72.2 | - | - | - | - | - | - | 1.0 | 9.1 | 0.2 (0.3) | 2 | 18.2 | 0.5 | - | - | - | - | - | 11 | 100.0 | 409 |  |
| 2023 16 | 2 | 66.7 | - | - | - | - | - | - | - | - | - | 1 | 33.3 | 0.3 | - | - | - | - | - | 3 | 100.0 | 398 |  |
| 2023 17 | 8 | 88.9 | 1 | 11.1 | 0.3 | - | - | - | - | - | - | - | - | - | - | - | - | - | - | 9 | 100.0 | 395 |  |
| 2023 18 | 8 | 88.9 |  |  | 0.0 | - | - | - | - | - | - | 1 | 11.1 | 0.3 | - | - | - | - | - | 9 | 100.0 | 386 |  |
| 2023 19 | 8 | 80 | 1 | 10.0 | 0.3 | - | - | - | - | - | - | 1 | 10.0 | 0.3 | - | - | - | - | - | 10 | 100.0 | 377 |  |
| 2023 20 | 6 | 85.7 | 1 | 14.3 | 0.3 | - | - | - | - | - | - | 0 | - | 0.0 | - | - | - | - | - | 7 | 100.0 | 367 |  |
| 2023 21 | 7 | 87.5 | - | - | - | - | - | - | - | - | - | 1 | 12.5 | 0.3 | - | - | - | - | - | 8 | 100.0 | 360 |  |
| 2023 22 | 6 | 100 | - | - | - | - | - | - | - | - | - | - | - | - | - | - | - | - | - | 6 | 100.0 | 352 |  |
| Not classified | 31 | 86.1 | 1 | 2.8 | 0.3 | 1 | 2.8 | 0.3 | - | - | - | 1 | 2.8 | 0.3 |  |  |  | 2 | 5.6 | 36 | 100.0 | 346 |  |
| **Total** | **747** | **65.7** | **178** | **15.7** | - | **74** | **6.5** | **-** | **6.0** | **0.5** | **-** | **109** | **9.6** | **-** | **10** | **0.9** | **-** | **13** | **1.1** | **1137** | **100.0** | 310 |  |

*CW. calendar week; ADV. adenovirus; RSV. respiratory syncytial virus; SARS-CoV-2. severe acute respiratory syndrome coronavirus 2
*If applicable, incidences corrected for sensitivity of MAK5 test (Supplementary Table 1; SARS-CoV-2=96,36%; RSV 93,81%; ADV 94,29%; Influenza A 94,96%; Influenza B 95,37%) given in parentheses. Corrected incidence was calculated as corrected cases (= number of detections / sensitivity) divided by the total number of active participants.*

Supplementary Table 6. **Co-detections during longitudinal ARI assessment from test receipt to May 2023 and at the point prevalence assessment in June 2023**

| *Co-detections* | *Calendar week* | *Detections during continuous ARI assessment N* | *Detections at*  *point prevalence  N* | *TOTAL     N* |
| --- | --- | --- | --- | --- |
| *ADV + Influenza A virus* | *2022 49* | *1* | *0* | *1* |
| *ADV + RSV* | *2022 50* | *2* | *0* | *2* |
|  | *2022 52* | *1* | *0* | *1* |
|  | *2023 01* | *2* | *0* | *2* |
|  | *2023 07* | *1* | *0* | *1* |
|  | *2023 08* | *1* | *0* | *1* |
|  | *2023 22* | *0* | *4* | *4* |
| *Influenza A- + /B virus + SARS-CoV-2* | *2023 01* | *1* | *0* | *1* |
| *Influenza A virus + RSV* | *2022 49* | *1* | *0* | *1* |
|  | *2022 50* | *2* | *0* | *2* |
|  | *2022 52* | *1* | *0* | *1* |
|  | *2023 9* | *1* | *0* | *1* |
| *Influenza A virus + SARS-CoV-2* | *2022 50* | *1* | *0* | *1* |
| *RSV + SARS-CoV-2* | *2022 49* | *1* | *0* | *1* |
|  | *2022 52* | *1* | *0* | *1* |
| *TOTAL* | *-* | *17* | *4* | *21* |

*ADV. Adenovirus; ARI, acute respiratory infection; RSV. respiratory syncytial virus; SARS-CoV-2. severe acute respiratory syndrome coronavirus 2*

Supplementary Table 7. **Reported symptoms by MAK5 test results per pathogen**

|  | **Negative test result** | | **SARS-CoV-2** | | **Influenza A virus** | | **Influenza B virus** | | **RSV** | | **ADV** | | **Total** | |  | | **Negative test result** | | | **SARS-CoV-2** | | **RSV** | | **ADV** | | **Total** | |  |
| --- | --- | --- | --- | --- | --- | --- | --- | --- | --- | --- | --- | --- | --- | --- | --- | --- | --- | --- | --- | --- | --- | --- | --- | --- | --- | --- | --- | --- |
|  | **Longitudinal assessment: December 2022 – May 2023**  **n [%]** | | | | | | | | | | | | | | |  | | **Point prevalence assessment: June 2023**  **n [%]** | | | | | | | | | | |
| **Reported symptoms**  Median (IQR) [range] | 3 (1-4)  [0-10] | | 4 (3-5)  [1-10] | | 5 (4-6)  [2-9] | | 4 (3-4)  [3-6] | | 4 (3-5)  [1-10] | | 3 (2-5)  [1-6] | | 3 (1-4)  [0-10] | |  | | 1 (1-5)  [0-5] | | | 3 (1-5)  [1-5] | | 0 (0-1)  [0-3] | | 0 (0-1)  [0-2] | | 1 (1-5)  [0-5] | |  |
| **No symptoms** | - | - | - | - | - | - | - | - | - | - | - | - | - | - |  | | 241 | | 85.5 | - | - | 14 | 63.6 | 3 | 75.0 | 258 | 83.2 |  |
| Rhinitis | 567 | 75.9 | 124 | 69.7 | 56 | 75.7 | 3 | 50.0 | 86 | 78.9 | 8 | 80.0 | 851 | 74.8 |  | | 21 | | 7.5 | 2 | 100. | 5 | 22.7 | - | - | 28 | 8.9 |  |
| Cough | 445 | 59.6 | 121 | 68.0 | 66 | 89.2 | 5 | 83.3 | 73 | 67.0 | 5 | 50.0 | 722 | 63.5 |  | | 5 | | 1.8 | - | - | 2 | 9.1 | - | - | 7 | 2.2 |  |
| Pharyngitis | 472 | 63.2 | 93 | 52.2 | 28 | 37.8 | 4 | 66.7 | 64 | 58.7 | 6 | 60.0 | 671 | 59.0 |  | | 10 | | 3.6 | 1 | 50.0 | 3 | 13.6 | - | - | 14 | 4.5 |  |
| Headache | 229 | 30.7 | 84 | 47.2 | 35 | 47.3 | 2 | 33.3 | 37 | 33.9 | 3 | 30.0 | 390 | 34.3 |  | | 2 | | 0.7 | 1 | 50.0 | 1 | 4.5 | - | - | 4 | 1.3 |  |
| Fever | 118 | 15.8 | 65 | 36.5 | 41 | 55.4 | 2 | 33.3 | 20 | 18.3 | 3 | 30.0 | 250 | 22.0 |  | | - | | - | 1 | 50.0 | - | - | - | - | 1 | 0.3 |  |
| Myalgia | 105 | 14.1 | 63 | 35.4 | 32 | 43.2 | 1 | 16.7 | 22 | 20.2 | 1 | 10.0 | 224 | 19.7 |  | | - | | - | 1 | 50.0 | - | - | - | - | 1 | 0.3 |  |
| Asthenia | 121 | 16.2 | 48 | 27.0 | 17 | 23.0 | 1 | 16.7 | 28 | 25.7 | 1 | 10.0 | 217 | 19.1 |  | | 5 | | 1.8 | - | - | 1 | 4.5 | - | - | 6 | 1.9 |  |
| Dyspnea | 48 | 6.4 | 16 | 9.0 | 6 | 8.1 | 1 | 16.7 | 8 | 7.3 | - | - | 79 | 6.9 |  | | 1 | | 0.4 | - | - | - | - | - | - | 1 | 0.3 |  |
| Conjunctivitis | 64 | 8.6 | 4 | 2.2 | 10 | 13.5 | - | - | 9 | 8.3 | - | - | 87 | 7.7 |  | | 1 | | 0.4 | - | - | 1 | 4.5 | - | - | 2 | 0.6 |  |
| Chills | 32 | 4.3 | 20 | 11.2 | 15 | 20.3 | - | - | 11 | 10.1 | - | - | 79 | 6.9 |  | | - | | - | - | - | - | - | - | - | - | - |  |
| Ear involvement | 32 | 4.3 | 6 | 3.4 | 6 | 8.1 | - | - | 6 | 5.5 | 1 | 10.0 | 51 | 4.5 |  | | 1 | | 0.4 | - | - | - | - | - | - | 1 | 0.3 |  |
| Hoarseness | 27 | 3.6 | 11 | 6.2 | 3 | 4.1 | 1 | 16.7 | 5 | 4.6 | - | - | 48 | 4.2 |  | | 1 | | 0.4 | - | - | - | - | - | - | 1 | 0.3 |  |
| Sputum production | 23 | 3.1 | 6 | 3.4 | 3 | 4.1 | - | - | 6 | 5.5 | - | - | 38 | 3.3 |  | | - | | - | - | - | - | - | - | - | - | - |  |
| Anosmia | 18 | 2.4 | 9 | 5.1 | 2 | 2.7 | - | - | 6 | 5.5 | 1 | 10.0 | 37 | 3.3 |  | | - | | - | - | - | - | - | - | - | - | - |  |
| Ageusia | 19 | 2.5 | 9 | 5.1 | 4 | 5.4 | - | - | 2 | 1.8 | - | - | 35 | 3.1 |  | | - | | - | - | - | - | - | - | - | - | - |  |
| GIT symptoms | 12 | 1.6 | 10 | 5.6 | 3 | 4.1 | - | - | 4 | 3.7 | 1 | 10.0 | 30 | 2.6 |  | | 1 | | 0.4 | - | - | - | - | - | - | 1 | 0.3 |  |
| Tonsilitis | 14 | 1.9 | 8 | 4.5 | 2 | 2.7 | - | - | 2 | 1.8 | - | - | 26 | 2.3 |  | | - | | - | - | - | 1 | 4.5 | 1 | 25.0 | 2 | 0.6 |  |
| Vertigo | 11 | 1.5 | 5 | 2.8 | 3 | 4.1 | - | - | 2 | 1.8 | - | - | 21 | 1.8 |  | | 1 | | 0.4 | - | - | - | - | - | - | 1 | 0.3 |  |
| Sternutation | 12 | 1.6 | - | - | 1 | 1.4 | - | - | 6 | 5.5 | - | - | 19 | 1.7 |  | | 1 | | 0.4 | - | - | - | - | - | - | 1 | 0.3 |  |
| Lymphadenopathy | 11 | 1.5 | 1 | 0.6 | - | - | - | - | 2 | 1.8 | - | - | 14 | 1.2 |  | | - | | - | - | - | - | - | - | - | - | - |  |

*ADV. adenovirus; COPD. chronic obstructive pulmonary disease; COVID-19. coronavirus disease 2019; GIT. gastrointestinal; HIV. human immunodeficiency virus; INF. infection; IQR. interquartile range; RSV. respiratory syncytial virus; SARS-CoV-2. severe acute respiratory syndrome coronavirus 2; yrs. years; VACC. vaccination*

Supplementary Table 8. **Proportional comparisons in participants with acute respiratory infection (ARI)***

|  | **SARS-CoV-2** | | **Influenza A virus** | | **RSV** | | **p value**** |
| --- | --- | --- | --- | --- | --- | --- | --- |
| **Sex** |  | |  | |  | | 0.204 |
| Woman | 114 | 65.5% | 36 | 53.7% | 60 | 62.5% |  |
| Man | 60 | 34.5% | 31 | 46.3% | 36 | 37.5% |  |
| **Age** |  |  |  |  |  |  | <0.001 |
| 18-29 | 14 | 8.0% | 9 | 13.4% | 12 | 12.5% |  |
| 30-39 | 36 | 20.7% | 32 | 47.8% | 27 | 28.1% |  |
| 40-49 | 40 | 23.0% | 11 | 16.4% | 26 | 27.1% |  |
| 50-59 | 42 | 24.1% | 13 | 19.4% | 20 | 20.8% |  |
| 60-69 | 36 | 20.7% | 0 | 0.0% | 9 | 9.4% |  |
| 70+ | 6 | 3.4% | 0 | 0.0% | 1 | 1.0% |  |
| **Reported symptoms** |  |  |  |  |  |  |  |
| Cough | 119 | 68.4% | 61 | 91.0% | 67 | 69.8% | 0.002 |
| Pharyngitis | 92 | 52.9% | 26 | 38.8% | 57 | 59.4% | 0.017 |
| Fever | 64 | 36.8% | 40 | 59.7% | 19 | 19.8% | <0.001 |
| Rhinitis | 120 | 69.0% | 49 | 73.1% | 76 | 79.2% | 0.263 |
| Dyspnea | 16 | 9.2% | 5 | 7.5% | 7 | 7.3% | 0.867 |
| Conjunctivitis | 3 | 1.7% | 8 | 11.9% | 6 | 6.3% | 0.002 |
| Anosmia | 9 | 5.2% | 1 | 1.5% | 4 | 4.2% | 0.581 |
| Ageusia | 9 | 5.2% | 3 | 4.5% | 1 | 1.0% | 0.227 |
| Myalgia | 63 | 36.2% | 29 | 43.3% | 17 | 17.7% | 0.001 |
| Headache | 83 | 47.7% | 32 | 47.8% | 33 | 34.4% | 0.108 |
| Asthenia | 48 | 27.6% | 16 | 23.9% | 28 | 29.2% | 0.789 |
| Hoarseness | 11 | 6.3% | 2 | 3.0% | 4 | 4.2% | 0.673 |
| Chills | 20 | 11.5% | 13 | 19.4% | 9 | 9.4% | 0.133 |
| GIT symptoms | 10 | 5.7% | 4 | 6.0% | 5 | 5.2% | 0.945 |
| Ear involvement | 6 | 3.4% | 4 | 6.0% | 3 | 3.1% | 0.607 |
| Vertigo | 5 | 2.9% | 3 | 4.5% | 2 | 2.1% | 0.696 |
| Sternutation | 0 | 0.0% | 1 | 1.5% | 6 | 6.3% | 0.001 |
| Lymphadenopathy | 1 | 0.6% | 0 | 0.0% | 2 | 2.1% | 0.293 |
| Tonsilitis | 8 | 4.6% | 2 | 3.0% | 2 | 2.1% | 0.634 |
| Sputum production | 6 | 3.4% | 3 | 4.5% | 6 | 6.3% | 0.528 |
| **Underlying conditions** |  |  |  |  |  |  |  |
| Hypertension | 24 | 13.8% | 6 | 9.0% | 13 | 13.5% | 0.640 |
| Coronary heart disease | 2 | 1.1% | 0 | 0.0% | 2 | 2.1% | 0.656 |
| Heart failure | 1 | 0.6% | 0 | 0.0% | 1 | 1.0% | 1.000 |
| Asthma/COPD | 18 | 10.3% | 3 | 4.5% | 11 | 11.5% | 0.315 |
| Chronic liver disease | 1 | 0.6% | 0 | 0.0% | 0 | 0.0% | 1.000 |
| Chronic kidney disease | 0 | 0.0% | 0 | 0.0% | 2 | 2.1% | 0.117 |
| ≥20 kg overweight | 17 | 9.8% | 6 | 9.0% | 8 | 8.3% | 0.968 |
| Diabetes mellitus | 4 | 2.3% | 1 | 1.5% | 0 | 0.0% | 0.404 |
| HIV | 4 | 2.3% | 1 | 1.5% | 1 | 1.0% | 0.865 |
| Active cancer (≥2 yrs) | 1 | 0.6% | 0 | 0.0% | 1 | 1.0% | 1.000 |
| Epilepsy | 2 | 1.1% | 2 | 3.0% | 0 | 0.0% | 0.175 |
| Chronic GIT disease | 3 | 1.7% | 0 | 0.0% | 3 | 3.1% | 0.344 |
| Serious musculoskeletal disease | 3 | 1.7% | 1 | 1.5% | 3 | 3.1% | 0.693 |
| Mental illness | 10 | 5.7% | 3 | 4.5% | 7 | 7.3% | 0.785 |
| History of stroke | 0 | 0.0% | 0 | 0.0% | 1 | 1.0% | 0.477 |

*COPD, chronic obstructive pulmonary disease; GIT, gastrointestinal; HIV, human immunodeficiency virus; RSV, respiratory syncytial virus; SARS-CoV-2, severe acute respiratory syndrome coronavirus 2*
*Only single SARS-CoV-2, Influenza virus A and RSV detections included (no co-infections); influenza B virus and ADV excluded due to small number; only symptomatic participants (longitudinal ARI assessment)

**3. Supplementary Figures**

Supplementary Figure 1. **Multipathogen antigen-based lateral flow rapid test (MAK5, BioTeke Corporation (Wuxi) Co., Ltd., Wuxi, Jiangsu, China): (A) positive for RSV (B) contents of parcel including test instructions and (C) certificate, swab and test liquid**


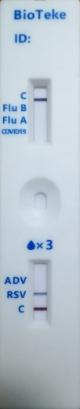

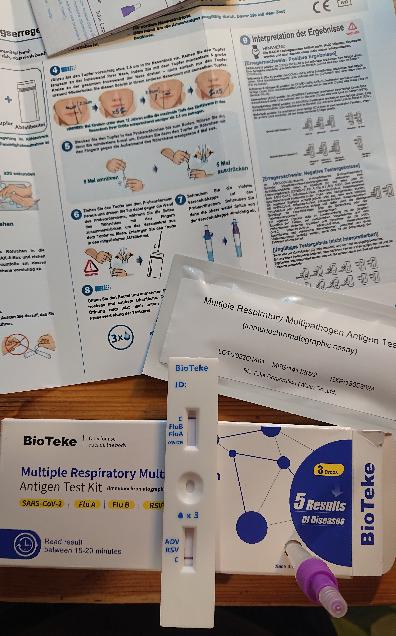

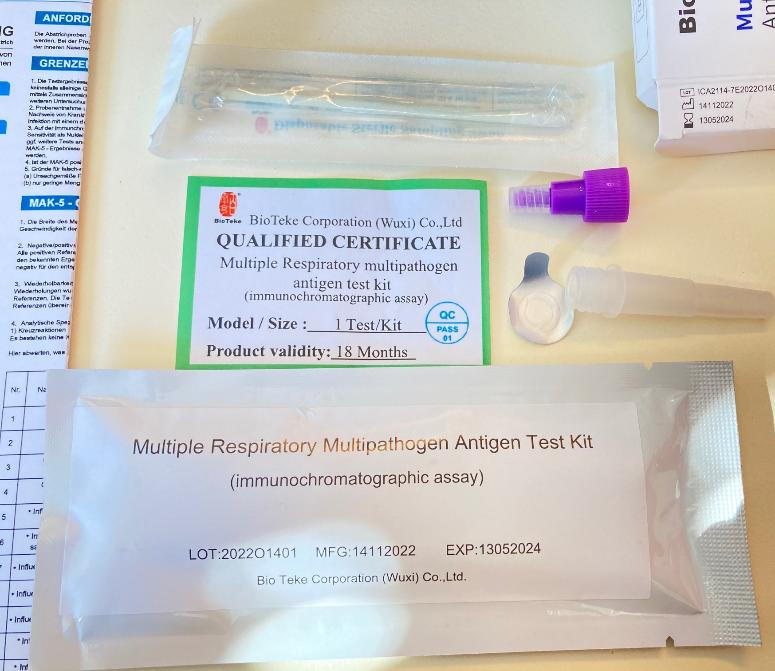


A

B

C

*ADV, adenovirus; Flu A, Influenza A virus; Flu B, Influenza B virus; C, control; ID, identification number; RSV, respiratory syncytial virus; SARS-CoV-2, severe acute respiratory syndrome coronavirus 2*

Supplementary Figure 2. **MAK5 test results by age group December 2022 – May 2023**

*ADV, adenovirus; RSV, respiratory syncytial virus; SARS-CoV-2, severe acute respiratory syndrome coronavirus 2; MAK5, multiple respiratory multipathogen antigen test kit*

Supplementary Figure 3. **MAK5 test results by age group in percentages December 2022 – May 2023**

*ADV, adenovirus; RSV, respiratory syncytial virus; SARS-CoV-2, severe acute respiratory syndrome coronavirus 2; MAK5, multiple respiratory multipathogen antigen test kit*

Supplementary Figure 4. **Reported symptoms by participants with negative test result December 2022 – May 2023**

*Total participants with negative test result n=717*

Supplementary Figure 5. **Reported symptoms by participants with SARS-CoV-2 detection December 2022 – May 2023**

*SARS-CoV-2, severe acute respiratory syndrome coronavirus 2; Total participants with SARS-CoV-2 detection n=178*

Supplementary Figure 6. **Reported symptoms by participants with RSV detection December 2022 – May 2023**

*RSV, respiratory syncytial virus; Total participants with RSV detection n=111*

Supplementary Figure 7. **Reported symptoms by participants with influenza A virus detection December 2022 – May 2023**

*Total participants with Influenza A virus detection n=75*

Supplementary Figure 8. **Reported symptoms by participants with influenza B virus detection December 2022 – May 2023**

*Total participants with Influenza B virus detection n=6*

Supplementary Figure 9. **Reported symptoms by participants with ADV detection December 2022 – May 2023**

*ADV, adenovirus; Total participants with ADV detection n=11*

Supplementary Figure 10. **Point prevalence assessment June 1, 2023**

*ADV, adenovirus; RSV, respiratory syncytial virus; SARS-CoV-2, severe acute respiratory syndrome coronavirus 2*
